# Long-Acting Injectable Antipsychotics: Are They the Missing Link in Early Psychosis Treatment?

**DOI:** 10.1192/j.eurpsy.2025.2086

**Published:** 2025-08-26

**Authors:** I. M. Lopes, D. Seabra, G. Santos, N. Ramalho, T. Coelho Rocha, J. Alves Leal, J. F. Cunha, J. Moura, D. Santos, A. Garcia, M. Rosa, C. Pires, L. Lopes, M. Cameira

**Affiliations:** 1 Psychiatry, ULS Arco Ribeirinho; 2 Psychiatry, USL Arco Ribeirinho; 3 Psychiatry, ULS Barreiro, Barreiro; 4 Psychiatry, ULS Alto Minho, Viana do Castelo; 5 Psychiatry, ULS São José, Lisboa, Portugal

## Abstract

**Introduction:**

Research into early interventions following a first episode of psychosis (FEP) has enabled a focused approach on prognostic-modifying factors. Among these, poor medication adherence contributes to relapse, as well as cognitive and functional deterioration. Several studies report discontinuation rates of oral antipsychotics (OAPs) after FEP at 70%, regardless of the prescribed OAP. The early introduction of long-acting injectable antipsychotics (LAIAs) could present an alternative.

**Objectives:**

This study aims to review the efficacy of LAIAs in the early stages of psychosis and compare the most relevant international guidelines on this topic.

**Methods:**

**Methodology:** A non-systematic literature review using the keywords “long-acting injectable” and “first episode psychosis,” limited to articles published in English and Portuguese in the last 10 years from the PubMed®/MEDLINE® database, and clinical practice guidelines on psychosis, schizophrenia, and FEP from NICE, APA, and RANZCP.

**Results:**

Despite frequent selection biases (such as reserving LAIAs for patients with worse prognostic factors), significant benefits of LAIAs over OAPs in preventing hospitalization and relapse during the early phases of psychosis are consistently reported. LAIAs reduce non-adherence due to forgetfulness or reduced insight, while their different pharmacokinetics minimize withdrawal symptoms, drug interactions, and fluctuations in plasma concentration, enhancing tolerability. No second-generation LAIA was found to be clearly superior in terms of efficacy. Various guidelines recommend offering this treatment option early, favoring an informed and collaborative decision-making process. However, despite documented benefits in robust studies, they do not consider LAIAs as a first-line treatment.

**Conclusions:**

**Discussion/Conclusions:** Significant variations in the proportion of patients on LAIAs across countries suggest that factors other than efficacy may influence its use. Greater understanding of these factors could help identify potential barriers to optimal implementation. New evidence may prompt a review of the guidelines.

**Disclosure of Interest:**

None Declared

